# Body Complexion and Circulating Lipids in the Risk of TDP-43 Related Disorders

**DOI:** 10.3389/fnagi.2022.838141

**Published:** 2022-03-25

**Authors:** Noelia Esteban-García, Luis C. Fernández-Beltrán, Juan Miguel Godoy-Corchuelo, Jose L. Ayala, Jordi A. Matias-Guiu, Silvia Corrochano

**Affiliations:** ^1^Neurological Disorders Group, Hospital Clínico San Carlos, Instituto de Investigación Sanitaria San Carlos (IdISSC), Madrid, Spain; ^2^Department of Computer Architecture and Automation, Universidad Complutense de Madrid, Madrid, Spain

**Keywords:** amyotrophic lateral sclerosis, frontotemporal dementia, lipid metabolism, Mendelian randomization, TDP-43 related disorders

## Abstract

**Objective:**

Frontotemporal dementia (FTD) and amyotrophic lateral sclerosis (ALS) are two distinct degenerative disorders with overlapping genetics, clinical manifestations, and pathology, including the presence of TDP-43 aggregates in nearly 50% of patients with FTD and 98% of all patients with ALS. Here, we evaluate whether different genetically predicted body lipid metabolic traits are causally associated with the risk of FTD with TDP-43 aggregates, compare it to their causal role in the risk of ALS, and identify genetic variants shared between these two TDP43 related disorders in relation to lipid metabolic traits.

**Methods:**

We conducted two-sample Mendelian randomization analyses (2SMR) to evaluate the causal association of 9 body complexion and 9 circulating lipids traits with the risk of FTD with TDP-43 aggregates and the risk of ALS. The inverse-variance weighted method was the primary analysis, followed by secondary sensitive analyses. We then looked for common genetic variants between FTD and ALS in relation to lipid metabolic traits.

**Results:**

Genetically increased trunk-predicted mass, fat-free mass, and higher circulating triglycerides levels were suggestively associated with a higher risk of FTD with TDP-43 aggregates. Circulating lipids, mainly LDL cholesterol, were causally associated with a higher risk of ALS. We identified two genetic variants, *EIF4ENIF1 and HNRNPK*, in relation to body complexion and circulating lipids shared between FTD with TDP-43 aggregates and ALS.

**Conclusion:**

This work provides evidence that body complexion and circulating lipids traits impact differentially on the risk of FTD and ALS, suggesting new and specific interventional approaches in the control of body lipid metabolism for FTD and ALS, and identified *HNRNPK* as a potential link between circulating lipids levels and these disorders.

## Introduction

Frontotemporal lobar degeneration (FTLD) encompasses a spectrum of severe neurodegenerative disorders with several degrees of behavioral, language, cognitive, and motor symptoms. Frontotemporal dementia (FTD) is characterized by neuronal degeneration of the frontal and anterior temporal lobes of the brain. It is the second most common cause of early onset dementia (age < 65) and the third in patients > 65 years (Seltman and Matthews, 2012; Coyle-Gilchrist et al., 2016; Manzoni and Ferrari, 2021). Currently, there is no treatment for FTD.

Frontotemporal dementia is mainly sporadic. However, genetics plays a key role in up to 30% of the cases (Manzoni and Ferrari, 2021). So far, more than 20 genes involved in the development of FTLD have been identified. The protein aggregates, being the largest group characterized by positive inclusions of TAR DNA binding protein 43 (TDP-43), e.g., FTLD TDP subtype, are a hallmark of these disorders. Interestingly, in amyotrophic lateral sclerosis (ALS), another degenerative debilitating disorder, TDP-43 aggregates are found in up to 98% of the cases studied (Neumann et al., 2006; Geser et al., 2009). ALS is a neurodegenerative fatal disorder characterized by the loss of upper and lower motor neurons of the brain and spinal cord leading to muscle atrophy and death (Kiernan et al., 2011). ALS and FTD are two ends of a spectrum of disorders with several mixed intermediate forms (Ferrari et al., 2011). There are some genes that can cause both ALS or FTD or mixed forms (DeJesus-Hernandez et al., 2011; Xi et al., 2014; Karch et al., 2018; Raffaele et al., 2019). There might be some missing genetics that could help to shed light not only on these complex disorders, but also on the influence of environmental risk factors. Especially, the weight and circulating lipids are complex traits that have been previously associated with ALS (Dupuis et al., 2008; Paganoni et al., 2011), and to a much lesser extent with FTD. The weight seems to be delineating the clinical form along the spectrum, with the smaller weight at the ALS end and the highest weight in the FTD end of the spectrum (Ahmed et al., 2014). Association studies have revealed that patients with FTD have lipid metabolism alterations (Kim et al., 2018a; Ahmed et al., 2019). This highlights the need to deepen in their association and potential causal relation since adiposity and metabolic traits have become a global health problem. Additionally, the identification of genetic variants that might be influencing the clinical form within the ALS/FTD spectrum of disorders could help predict the impact of these lipid metabolic risk factors and assist with the stratification of patients within the spectrum.

**FIGURE 1:**
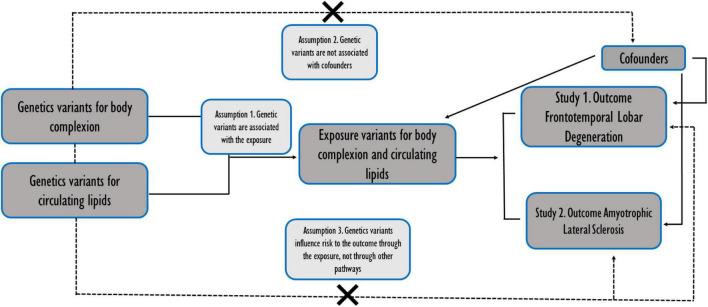
Schematic representation of the two independent studies conducted in parallel. Assumptions of a two-sample Mendelian randomization analyses between the exposures (body complexion and circulating lipids traits) and the outcomes (the risk of frontotemporal dementia TDP43 subtype and amyotrophic lateral sclerosis). Broken lines represent potential pleiotropic or direct causal effects between variables that would violate Mendelian randomization assumptions.

Here, we hypothesized that some of the genetic variants that impact in body lipid metabolic traits could be influencing the development of particular clinical forms of TDP-43 proteionopathies within the spectrum of disorders of FTD and ALS. To assess this hypothesis, we used an analytical tool known as Mendelian randomization analysis (MR) of 2 samples (2SMR) (Hemani et al., 2018), which allows for the identification of overlapping genetic variants between several risk factors and the disease of interest. Thus, we performed a 2SMR study using the most up-to-date GWAS summary data related to lipid metabolism in two main blocks: (i) body complexion; and, (ii) circulating lipids, with an FTLD TDP subtype GWAS summary data, and, in parallel, another study with GWAS of ALS (Figure 1). After the comparison of the two studies, we obtained all the significant SNPs in FTLD TDP subtype and in ALS, and we identified two genetic variants in relation to lipid metabolic traits shared between the two diseases of interest.

## Materials and Methods

### Genome-Wide Association Studies Summary Data for Body Complexion and Circulating Lipids (Exposures)

We used summary population-level data to conduct the method of 2SMR to evaluate the possible causal relationship between different variables related to body lipid metabolism and FTLD TDP subtype, and compared it to their role in ALS, a related TDP-43 proteinopathy. We conducted a Pubmed search as well as the MR-base catalog search of the most recent GWAS studies (until December 2020) for lifelong lipid metabolism traits, in particular those related to body composition, body shape and lipid levels in blood. We selected GWAS datasets using the following criteria: (i) GWAS with the most recent publication date; (ii) GWAS that have not been used in this type of analysis before; (iii) those with larger sample sizes; and, (iv) GWAS that have the data curated and were in the MR base catalog.

The exposure traits were separated into two groups: (i) Body complexion and adiposity, and (ii) circulating lipids. In the body complexion and adiposity group we included 9 traits: “Extreme body mass index,” “body mass index” (BMI), “waist-to-hip ratio” (WHR), “body fat percentage” (BF%), “trunk predicted mass,” “trunk fat-free mass,” “appendicular lean mass,” “impedance whole body,” “whole Body fat mass.” In the circulating lipids group, we included 9 traits: “Total triglycerides” (TG), “total cholesterol”(TC), “LDL cholesterol” (LDL), “HDL cholesterol” (HDL), “linoleic acid,” “docosahexaenoate” (DHA), “omega 3-fatty acid,” “omega 6-fatty acid,” “total fatty acids.” The characteristics of the selected GWAS are detailed in Supplementary Table 1.

### Genome-Wide Association Analysis Data for Frontotemporal Dementia and Amyotrophic Lateral Sclerosis (Outcomes)

We used the summary statistics from a GWAS of frontotemporal lobar degeneration with TDP-43 inclusions (FTLD TDP subtype) from 2010, with 3,020 cases in the study (Van Deerlin et al., 2010). We also ran the same study for the outcome ALS, in this case using the GWAS from 2018, gathering an *N* of 80,610 with 20,806 cases and 59,804 controls, and 10,031,630 variants in the analysis (Nicolas et al., 2018). All the populations are of European ancestry (Supplementary Table 1).

### The Two-Sample Mendelian Randomization Analyses

We used an algorithm developed by Hemani et al. (2018) and implemented it into the TwoSampleMR R package (version 4.26). We used RStudio 1.4.1717 (2021) and R 4.1.1 software to perform the MR analyses. This data package offers a curated database containing summarized GWAS. This approach determines whether an exposure is causally associated with an outcome. Along these lines, the MR analysis carried out was supported by three assumptions: (i) That instrumental variables (IVs) are significantly associated with body complexion and adiposity and circulating lipids; (ii) IVs are not associated with confounding factors; (iii) the risk of the two outcomes, ALS or FTD, is only influenced by IVs (Figure 1).

**FIGURE 2:**
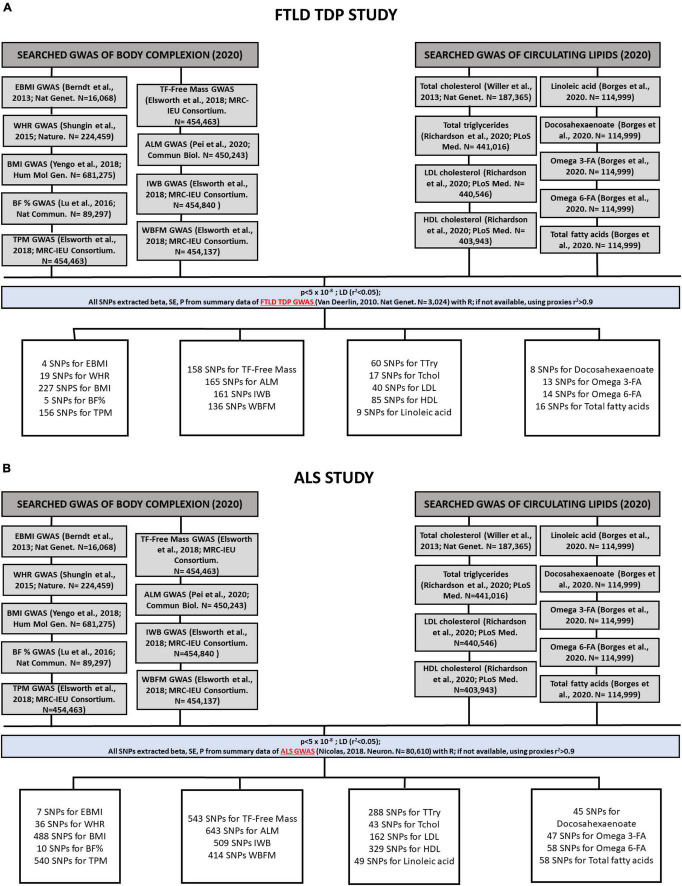
Flowchart followed by the process of identification and selection of genome-wide association studies (GWAS) and the SNPs used as instrumental variables (IVs) that have been included in the Mendelian randomization analyses of FTLD TDP subtype **(A)** and ALS **(B)**. In the body complexion group we included 9 traits: “Extreme body mass index,” “body mass index” (BMI), “waist-to-hip ratio” (WHR), “body fat percentage” (BF%), “trunk predicted mass,” “trunk fat-free mass,” “appendicular lean mass,” “impedance whole body,” “whole body fat mass.” In the circulating lipids group we included 9 traits: “Total triglycerides” (TG), “total cholesterol”(TC), “LDL cholesterol” (LDL), “HDL cholesterol” (HDL), “linoleic acid,” “docosahexaenoate” (DHA), “omega 3-fatty acid,” “omega 6-fatty acid,” and “total fatty acids.”

For each of the individual 2SMR analyses performed, the IVs used for each trait were identified. This identification is based on the odds ratios per allele (beta) and standard estimates (SE) for all single nucleotide polymorphisms (SNPs), using a statistically driven approach that identifies SNPs that are significantly associated with exposure, setting the threshold *p*-values of < 5 × 10^–8^. The applied algorithm then extracts those SNPs from the outcome (from FTLD TDP subtype and ALS GWAS datasets in this case), thereby selecting the SNPs found in both the exposure and outcome datasets. For the SNPs present in the exposure that are not present in the outcome data, it applies a clustering method using proxies (r_2_ > 0.9). With all these SNPs, a harmonization of the data sets is carried out to ensure that the effect of the size of the SNPs is homogenized. The total number of IVs used on each of the 2SMR analyses conducted for FTD and ALS are shown in Figures 2A,B, respectively.

The causal association between the exposures (the lipid metabolism traits) and the outcome (FTD or ALS) was primarily assessed by the inverse variance weighted (IVW) method, and the traits were considered to have an effect when the *p*-value was < 0.05. Multiple comparisons were corrected using the Bonferroni-corrected method. The IVW method has some assumptions on the independence of the genetic variants, assuming no horizontal pleiotropy that might be influencing the relation by other pathways (Burgess et al., 2013). As such, we used other sensitivity methods that account for pleiotropy to support the findings, including the MR-Egger regression, the Simple median, and the weighted median. To ensure the robustness of the significance of our results, we performed a leave-a-SNP-out analysis to detect the heterogeneity of the analyses.

We used the scatter plot to visualize how each SNP from the exposure used in the analysis was associated with the outcome, with a potential representation of the causal effect estimation of each of the SNPs.

### Identification of Shared Body Lipid Metabolisms Genetic Variants in Frontotemporal Dementia and Amyotrophic Lateral Sclerosis

From all of the 2SMR analyses performed, we extracted all the SNPs from body complexion and circulating lipid levels that were significantly associated with FTD TDP subtype, and we did the same for all the SNPs significantly associated with ALS, thus creating two lists of putative genetic variants influencing those two diseases, separating them by the type of trait they belong to (body complexion or circulating lipids). We then evaluated specificities and communalities of the SNPs between the two outcomes (FTD and ALS) using Venn diagrams. We used bioinformatics open resources as tools to examine the biological function of those SNPs, including dsSNP, GnomAD, ClinVar, and functional biological interactions using String.

## Results

Our main aim was to identify the genetically determined causal role of body lipid metabolism on the risk of FTD. In particular, we used the FTD with TDP-43 inclusion subtype as it is the pathological form that is more associated with ALS in the spectrum of disorders. Thus, we also ran the same study in parallel on ALS so that we could then compare similarities and specificities of the potential genetic influences of body lipid metabolism on the risk of these two disorders (Figure 1). For each disease (outcome), FTLD TDP subtype (Figure 2A) or ALS (Figure 2B), we analyzed the causal effect of a total of 9 traits included in the category of body complexion and another 9 traits that fall in the category of circulating lipids.

### Two-Sample Mendelian Randomization Analysis of Body Composition and Circulating Lipids on the Risk of Frontotemporal Dementia TAR DNA Binding Protein 43 Subtype

**FIGURE 3:**
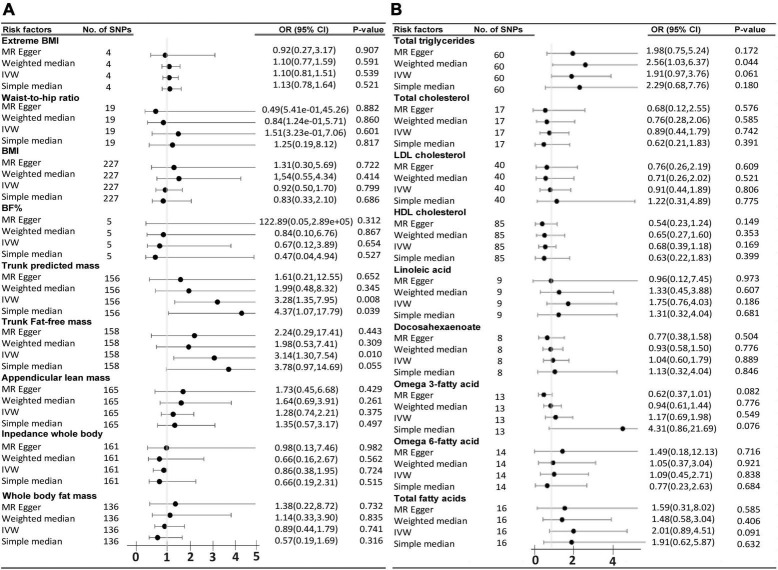
Odds ratio (OR) and forest plot for the causal association with the risk of frontotemporal dementia with TDP43 aggregates by, **(A)** Body complexion traits, and **(B)** Circulating lipids traits. BMI, body mass index; BF%, body fat percentage; CI, confidence interval; IVW, inverse variance weighted; SNPs, single nucleotide polymorphisms.

In relation to FTLD TDP subtype, among the group of 9 traits that fall in the category of the body complexion, the trunk-predicted mass (*p* = 0.008, OR = 3.27, 95% CI = 1.35–7.95) and the trunk-predicted fat-free mass (*p* = 0.01, OR = 3.14, 95% CI = 1.30–7.54) are suggested to increase the risk of FTD by the IVW method. The simple median method was able to sustain those results, especially in the case of trunk-predicted mass (*p* = 0.039), and close but not significant for the trunk-predicted fat-free mass (*p* = 0.055). Unfortunately, the results were not supported by the MR-Egger method (Figure 3A). A list of all the SNPs used in the analysis of the 9 traits on FTD are displayed in Supplementary Tables 2–10. Regarding the circulating lipid levels group, none of the 9 traits analyzed were significantly found to be genetically causally associated with FTD by the IVW method (Figure 3B). Genetically predicted “total triglycerides” showed a trend to be causally associated with FTD by the IVW method (*p* = 0.06, OR = 1.9, 95% CI = 0.96–3.7) and it was significantly associated by the weighted median method (*p* = 0.04, OR = 2.5, 95% CI = 1.02–6.36). A list of all the SNPs (IVs) used in the analysis of the 9 circulating lipid traits on FTD is displayed in the Supplementary Tables 11–19.

### Two-Sample Mendelian Randomization Analysis of Body Composition and Circulating Lipids on the Risk of Amyotrophic Lateral Sclerosis

**FIGURE 4:**
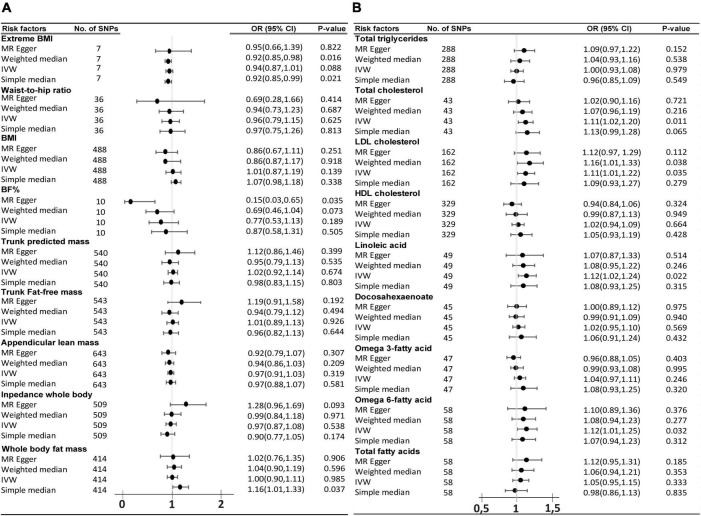
Odds ratio (OR) and forest plot for the causal association with the risk of ALS by, **(A)** body composition and, **(B)** circulating lipids. BMI, body mass index, BF%, body fat percentage, CI, confidence interval, IVW, inverse variance weighted, SNPs, single nucleotide polymorphisms.

The same study was conducted in parallel with ALS as the outcome. The primary analysis of IVW method did not find any significant causal association of the 9 body complexion traits with ALS (Figure 4A). The “extreme body mass index” (Extreme BMI) was found to be significant by the weighted median (*p* = 0.016, OR = 0.92, 95% CI = 0.85–0.98) and by simple median (*p* = 0.02, OR = 0.91, 95% CI = 0.85–0.99) methods, but the analysis was not rigorously significant by the IVW method (*p* = 0.08, OR = 0.93, 95% CI = 0.87–1.00). The “body fat percentage” (BF%) trait also presented a trend of potential inverse causal effect on ALS found significant by the MR-Egger method (*p* = 0.035, OR = 0.15, 95% CI = 0.03–0.65), consistent with a previous analysis by Zhang et al. (2020), although none of the other sensitive analyses supported those findings. The leave-one-out analysis of BF% identified the SNP rs6857 to be driving much of the effect, which is consistent with the previous analysis (Zhang et al., 2020). The leave-one-out analysis of extreme BMI identified the SNP rs11127483 to be driving much of the effect. Thus, the 1-SD increase in extreme BMI and in BF% is not rigorously associated with a lower risk of ALS, but more data and experiments are needed to clarify and support those results. A list of all the SNPs used in these analyses can be found in Supplementary Tables 2–10. From the 9 circulating lipids traits, we found that “total cholesterol” (*p* = 0.011, OR = 1.11, 95% CI = 1.02–1.2), “LDL cholesterol” (*p* = 0.034, OR = 1.11, 95% CI = 1.01–1.21), “linoleic acid” (*p* = 0.022, OR = 1.12, 95% CI = 1.016–1.24), and “omega-6 fatty acid” (*p* = 0.033, OR = 1.12, 95% CI = 1.00–1.25) were causally associated to ALS by the primary method IVW (Figure 4B). Out of the four blood lipid traits, only the “LDL cholesterol” was found supported significant by the weight median (*p* = 0.037, OR = 1.15, 95% CI = 1.00–1.32) and the weight mode (*p* = 0.015, OR = 1.16, 95% CI = 1.03–1.31) sensitive analysis. The list of IVs used in these analyses can be found in Supplementary Tables 11–19. The leave-one-out analysis showed no evidence of outlier SNPs.

**FIGURE 5:**
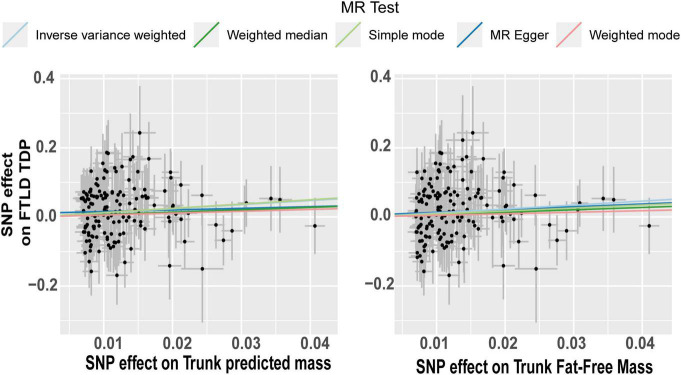
Scatterplot of single nucleotide polymorphism (SNP) potential effects on trunk mass vs. frontotemporal lobar degeneration with TDP43. The slope of each line corresponds to the estimated Mendelian randomization (MR) effect per method. Significant changes in SNPs related to predict trunk mass and trunk fat-free mass are associated as a risk factor in FTLD TDP subtype.

**FIGURE 6:**
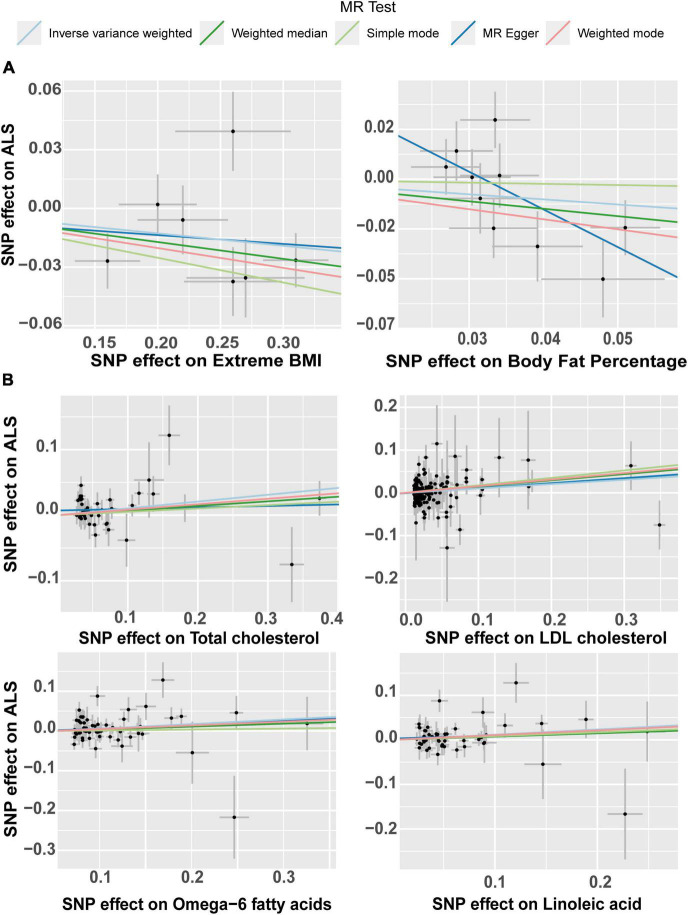
Scatter plot of single nucleotide polymorphism (SNP) potential effects on body lipid metabolic traits vs. amyotrophic lateral sclerosis (ALS). The slope of each line corresponds to the estimated Mendelian randomization (MR) effect per method. **(A)** Scatter plots show the comparison of the SNPs effects on extreme BMI (body mass index) and body fat percentage (BF%) vs. ALS**. (B)** Scatterplots of single nucleotide polymorphism (SNP) potential effects on total cholesterol, LDL cholesterol, Omega-6 fatty acid and linoleic fatty acid, vs. amyotrophic lateral sclerosis (ALS). LDL, low density lipoprotein.

The effect of the individual SNPs on the trunk predicted mass and fat-free mass vs. the effect on FTD is shown in Figure 5. The effect of the SNPs on the extreme BMI and BF% vs. their effect on ALS is represented in a scatter plot in Figure 6A. The effect of circulating lipids (total cholesterol, LDL cholesterol, linoleic acid and omega 6 fatty acid) vs. their effect on ALS is shown in scatter plots in Figure 6B.

### Identification of Shared and Specific Single Nucleotide Polymorphisms in Frontotemporal Dementia TAR DNA Binding Protein 43 Subtype and Amyotrophic Lateral Sclerosis in Relation to Body Lipid Metabolism

**FIGURE 7:**
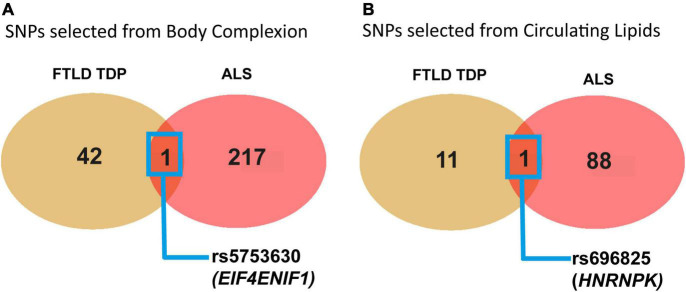
Graphical representation of the identification of common significant SNPs in relation to body complexion and circulating lipids shared between FTD TDP43 subtype and ALS. **(A)** Venn diagram showing the common significant SNPs between FTLD TDP subtype and ALS from the 9 traits in relation to the body complexion group. The only genetic variant found in common is rs5753630, with *EIF4ENIF1* as near gene. **(B)** Venn diagram showing the common significant SNPs between FTD and ALS from the 9 traits in relation to the circulating lipid traits. The only genetic variant found in common is rs696825 that falls in the 3′UTR of the *HNRNPK* gene.

In addition, we decided to look for the SNPs that showed a significant effect on the outcomes of FTLD TDP subtype and ALS and compared them with the idea that there could be some shared genetics regarding body lipid metabolism in these two disorders with pathological TDP43 hallmark overlap. Thus, we used all the results from the two studies and extracted the list of all the SNPs that were individually significantly associated with each of the outcomes, FTD and ALS. From the 9 traits in the group of body complexion (using the list of SNPs extracted from Supplementary Tables 2–10), we found a total of 43 SNPs that were significantly associated with FTD and a total of 218 SNPs significantly associated with ALS. We then compared the SNPs that were exclusively or commonly found between FTD and ALS, as shown in the Venn diagrams (Figure 7A), and identified only one SNP in common, rs5753630 [chr22:31465964 (GRCh38.p13)]. The rs5753630 variant falls on an intron region of the *EIF4ENIF1* gene (eukaryotic translation initiation factor 4E nuclear import factor 1), which encodes a nucleocytoplasmic shuttle protein involved in the regulation of translation initiation. We conducted further analysis of pathway enrichment to investigate if those 43 and 218 differential SNPs in FTD and ALS, respectively, are related to a biological function or a pathway. From the 43 SNPs specific of the FTLD TDP subtype and body complexion, we found that 32 SNPs have associated genes, and most of them have alternative splicing (data not shown).

Next, we performed the same analysis for the total of SNPs that were significantly associated with FTD and ALS from the study of circulating lipid traits (using the list of SNPs extracted from Supplementary Tables 11–19). We found 12 SNPs that were significant from the FTD study and 89 SNPs in the ALS study from all the 9 lipid traits analyzed. We then looked for any common SNPs between the two outcomes in relation to circulating lipids and identified one SNP, rs696825 [chr9:83968161 (GRCh38.p13)] (Figure 7B). The rs696825 variant falls on the 3‘ Prime UTR of the gene *HNRNPK* (heterogeneous nuclear ribonucleoprotein K), which encodes an hnRNP RNA binding protein involved in RNA processing. Interestingly, this protein has recently been found mislocalized in the cytoplasm and associated with FTLD pathology (Bampton et al., 2021). Remarkably, this protein seems to control the cytoplasmic aggregation of TDP43 (Moujalled et al., 2015). Finally, we run further enrichment analysis on the significant SNPs related to circulating lipid traits identified in the ALS and FTD studies. From the 89 SNPs of the ALS study, we found functional enrichment in the cholesterol homeostasis pathway (GO:42632). The list with all the SNPs used in this section can be found in Supplementary Table 20.

## Discussion

We conducted two independent studies in parallel to evaluate the effect of genetically predicted body complexion and circulating lipids on the risk of FTLD TDP subtype and on ALS and compared their effects in these two TDP-43 related diseases (Liscic et al., 2008) that are also part of a spectrum of disorders. We found that body trunk mass and triglycerides levels could be more relevant for the risk of FTLD TDP subtype, and on the contrary, the levels of LDL cholesterol, and possibly linoleic and omega 6 fatty acids, play a more important role in the risk of ALS. Moreover, we identified two genetic variants shared in the two studies conducted on FTD and ALS. These two variants are located in two genes that encode proteins with role in RNA metabolism which is a well-documented pathological mechanism operating in these disorders. Emerging evidence shows that lipid metabolic alterations as another important pathological mechanism contributing to these multifactorial complex disorders etiology (Burg et al., 2021; Fernández-Beltrán et al., 2021; Lee et al., 2021).

This is, to our knowledge, the first time that this type of MR analysis has been done to evaluate the genetically predicted causal association of these particular body complexions and circulating lipids traits on the risk of FTLD TDP subtype. We conducted the same study on ALS in parallel and compared the results of the two studies. There is a previous study using 2SMR with 5 different traits of lifelong adiposity on the risk of ALS, but our study encompasses a more extensive focus with 18 updated GWAS datasets that have not been used for ALS before, except for only two traits (BMI and extreme BMI), which are coincident with a previous study (Zhang et al., 2020). There is another interesting study using this approach for ALS using all the GWAS available at the time. In that study, they found that higher levels of LDL cholesterol in blood increases the risk of ALS (Bandres-Ciga et al., 2019). There is one more study which also identified that LDL cholesterol was causally associated with a higher risk of ALS (Zeng and Zhou, 2019). Here, we have been able to replicate those results even though we have used a different and updated GWAS data set for LDL cholesterol. We also found other suggestive associations that were not evaluated in those two previous studies. Thus, for both diseases, FTD and ALS, this work presents novel and interesting results.

We have shown, for the first time, that genetically predicted 1-SD increase in trunk mass and fat-free mass (lean mass) are suggestively associated with a higher risk of FTD (TDP43 subtype). More anthropometric measures should be conducted routinely in the clinic to verify this association. Body metabolic measurements done in observational association studies on FTLD have previously reported higher BMI and visceral fat mass in FTLD patients in comparison to healthy controls, as well as in patients with Alzheimer disease (Ahmed et al., 2019). Our analysis did not support a genetically causal association of BMI and fat mass with FTLD TDP subtype. Similarly, we could not find a significant causal relation of any of the 9 traits analyzed for body composition and anthropometric measures with the risk of ALS, except for a suggestive association of higher body fat percentage (BF%) and extreme BMI with lower risk of ALS, which would be in line with previous studies (Zhang et al., 2020).

In relation to the study of the causal association of blood circulating lipids on FTD, only the genetically predicted 1-SD increase triglycerides (TG) blood levels were suggestively associated with a higher risk of FTLD TDP subtype. Interestingly, previous blood lipid measurements in observational studies found hypertriglyceridemia in patients with behavioral variant FTD (bvFTD) vs. healthy controls and patients with Alzheimer disease (AD) (Kim et al., 2018a,b). Similarly, in another observational study comparing circulating lipid levels in bvFTD, ALS, as well as mixed forms of ALS-FTD, higher TG, and lower HDL cholesterol levels were found in all the groups compared to healthy controls (Ahmed et al., 2017; Kim et al., 2018a). None of the other circulating lipids traits, including HDL cholesterol, was causally associated with FTD in our study. We corroborated the previous causal association of 1-SD genetically predicted higher levels of LDL with the increased risk of ALS (Bandres-Ciga et al., 2019; Zeng and Zhou, 2019), even though we used an updated data source for the LDL trait. We found a novel suggested causal association with ALS in relation to circulating fatty acids, such as linoleic acid and the omega-6 fatty acid levels. It is well known that altered lipidemia is a clinical phenotype in patients with ALS (Dupuis et al., 2008; Paganoni et al., 2011), although this is the first time it is shown that these fatty acids could be also causally associated. Again, it is interesting to observe that not all the circulating lipids influence the risk of FTLD TDP subtype and ALS in the same way. These differences support the idea that different exposures (risk factors) might define the clinical manifestation of diseases with TDP-43 pathology.

The RNA processing disruption is a well-documented pathological mechanism in ALS and FTD. It is very interesting to observe that the two SNPs identified in common between ALS and FTLD TDP subtype, in relation to body complexion and circulating lipid levels, fall under genes related to RNA processing functions. It is very remarkable that even though the significantly associated circulating lipid traits were different between FTD and ALS, we could identify a common significant genetic variant in the *HNRNPK* gene in both the diseases. This gene is widely expressed in neuronal tissue and encodes for the hnRNP K protein. Similar to TDP-43, which is also a family member of hnRNP proteins, this protein binds to RNA and has a role in RNA processing and maturation. This protein is altered in models of ALS with TDP43 mutation (Moujalled et al., 2017), and most recently, it has been found mislocalized in patients with FTLD (Bampton et al., 2021). Curiously, it has been shown that hnRNP K phosphorylation controls the aggregation of TDP-43 in the cytosol (Moujalled et al., 2017). These findings suggest that HNRNPK could be a potential molecular causal link between the lipid metabolic alterations and ALS/FTD spectrum of disorders. Similarly, *HNRNPA1*, another gene that encodes a hnRNP protein that forms a functional complex with hnRNP K, affects the splicing and regulation of *HMGCR* (3-hydroxy-3-methylglutaryl-Coenzyme A reductase), the rate-limiting enzyme in the biosynthesis of cholesterol, impacting the intracellular cholesterol metabolism (Yu et al., 2014). Even more, TDP-43 regulates the splicing of hnRNP A1 (Deshaies et al., 2018). Further clinical and functional studies are needed to corroborate the potential role of HNRNPK in lipid homeostasis and TDP-43 pathology and understand whether there is a causal relation between altered body lipid metabolism and RNA processing in FTD and ALS.

There are some limitations in this study. First, all the MR studies might be subjected to pleiotropy since it is difficult to rule out linear relations between the risk factors and the diseases we studied. Second, we used European ancestry population mainly for the GWAS data we selected to reduce population stratification bias, but this might be difficult with the extension of these results to other genetic background populations. Third, all MR results are dependent on the SNPs used as instrumental variables in the particular trait data. Those could be different or updated with time, and thus it could potentially have a different result when performing the analysis after actualizing the data at a different time. In this particular case, we could identify new association trends between linoleic acid and omega 6 fatty acid levels with the risk of ALS by using updated databases and comparing with previous analysis results (Bandres-Ciga et al., 2019; Zeng and Zhou, 2019) that found no association. In general, the number of IVs used in the analysis with ALS is larger than those used in the analysis with FTD, partly due to the SNPs panels on each of the GWAS studies, which is more updated and extensive in the ALS GWAS (from 2018) than in the FTD GWAS study (from 2010), and also the sample size is much larger in the ALS GWAS (*n* = 80,610) compared to the FTD GWAS (*n* = 3,024). Therefore, all these parameters impact on the total number of SNPs detected that could then be used as IVs for the 2SMR analyses. Finally, here we limited the study to a specific FTLD subtype, and so the findings cannot be generalized to different clinical forms of FTLD. At the same time, this can also be perceived as a strength of the study since by performing the analysis with a particular FTLD type, we might be helping the identification of specific risk factors in the different subtypes, in this case the FTLD TDP subtype, supporting the stratification of patients and understanding in the clinic. Thus, the findings from the FTD with TDP-43 aggregates described in this study are very promising and opens the door for future analysis using new updated FTD GWAS databases, ideally with different FTLD forms GWAS datasets so that we could analyze the effect of all body lipid metabolic traits of interests in the different clinical forms of FTLD.

## Conclusion

We found that genetic variants associated with circulating lipids and body composition are differentially involved in the risk of FTLD TDP subtype and ALS. In this regard, our study suggests that higher LDL cholesterol and fatty acid levels could potentially have a causal role in the risk of ALS, but further experimental and clinical studies are needed to confirm these findings. Conversely, for FTD, the prevention and intervention strategies could be aimed to lower circulating triglycerides levels. These differences support the idea that different exposures (risk factors) might define clinical manifestation of diseases within the spectrum. These findings emphasize the need to study lipid metabolism in these complex disorders, and for more research on the causal role of circulating lipids the possibility to modify them for prevention or treatment, or even use them in the stratification of patients. We also identified HNRNPK as a potential candidate that could serve as a link between circulating lipids and ALS/FTD with TDP-43 pathology. Extensive experimental research is needed to confirm these findings.

## Data Availability Statement

The original contributions presented in the study are included in the article/Supplementary Material, further inquiries can be directed to the corresponding author/s.

## Author Contributions

JM-G and SC: conceptualization and funding acquisition. NE-G, LF-B, JG-C, and JA: methodology. NE-G, LF-B, and JA: software, validation, formal analysis, and data curation. NE-G, LF-B, JM-C, JA, JM-G, and SC: writing—review and editing. JA, JM-G, and SC: supervision. All authors have read and agreed to the published version of the manuscript.

## Conflict of Interest

The authors declare that the research was conducted in the absence of any commercial or financial relationships that could be construed as a potential conflict of interest. The reviewer RM declared a shared affiliation with one of the authors, JA to the handling editor at time of review.

## Publisher’s Note

All claims expressed in this article are solely those of the authors and do not necessarily represent those of their affiliated organizations, or those of the publisher, the editors and the reviewers. Any product that may be evaluated in this article, or claim that may be made by its manufacturer, is not guaranteed or endorsed by the publisher.
